# The effect of orthopaedic surgeons’ and interventional radiologists’ availability on the priority treatment sequence for hemodynamically unstable pelvic fractures: a survey of US Level I trauma centers

**DOI:** 10.1186/s13018-019-1417-1

**Published:** 2019-12-04

**Authors:** S. Jarvis, A. Orlando, B. Blondeau, K. Banton, C. Reynolds, G. M. Berg, N. Patel, R. Meinig, M. Carrick, D. Bar-Or

**Affiliations:** 1 Clinical Epidemiologist, ION Research, 383 Corona St. #319, Denver, CO 80218 USA; 2 Research Medical Center, 2316 East Meyer Blvd., Kansas City, MO 64132 USA; 3 University of Connecticut Hartford Hospital, Hartford, CT 06106 USA; 4 Swedish Medical Center, 501 E Hampden Ave., Englewood, CO 80113 USA; 5 Wesley Medical Center, 550 N. Hillside St., Wichita, KS 67214 USA; 6 Orthopaedic Trauma Surgeon, St. Anthony’s Hospital, 11600 West 2nd Place, Lakewood, CO 80228 USA; 7 Orthopaedic Trauma Surgeon, Penrose Hospital, 2222 North Nevada Ave., Colorado Springs, CO 80907 USA; 8 Medical City Plano, 3901 West 15th Street, Plano, TX 75075 USA

**Keywords:** Orthopaedic surgeons, Pelvic fracture management, Pelvic packing, Trauma, Angioembolization, Level I trauma center

## Abstract

**Background:**

Most guidelines recommend both pelvic packing (PP) and angioembolization for hemodynamically unstable pelvic fractures, however their sequence varies. Some argue to use PP first because orthopaedic surgeons are more available than interventional radiologists; however, there is no data confirming this.

**Methods:**

This cross-sectional survey of 158 trauma medical directors at US Level I trauma centers collected the availability of orthopaedic surgeons and interventional radiologists, the number of orthopaedic trauma surgeons trained to manage pelvic fractures, and priority treatment sequence for hemodynamically unstable pelvic fractures. The study objective was to compare the availability of orthopaedic surgeons to interventional radiologists and describe how the availability of orthopaedic surgeons and interventional radiologists affects the treatment sequence for hemodynamically unstable pelvic fractures. Fisher’s exact, chi-squared, and Kruskal-Wallis tests were used, alpha = 0.05.

**Results:**

The response rate was 25% (40/158). Orthopaedic surgeons (86%) were on-site more often than interventional radiologists (54%), *p* = 0.003. Orthopaedic surgeons were faster to arrive 39% of the time, and interventional radiologists were faster to arrive 6% of the time. There was a higher proportion of participants who prioritized PP before angioembolization at centers with above the average number (> 3) of orthopaedic trauma surgeons trained to manage pelvic fractures, as among centers with equal to or below average, *p* = 0.02. Arrival times for orthopaedic surgeons did not significantly predict prioritization of angioembolization or PP.

**Conclusions:**

Our results provide evidence that orthopaedic surgeons typically are more available than interventional radiologists but contrary to anecdotal evidence most participants used angioembolization first. Familiarity with the availability of orthopaedic surgeons and interventional radiologists may contribute to individual trauma center’s treatment sequence.

## Introduction

Mortality rates for patients with hemodynamically unstable pelvic fractures have been reported to be as high as 40% [1]. The optimal treatment sequence for patients with hemodynamically unstable pelvic fractures has yet to be determined; the most contentious treatment method is the application of pelvic packing (PP) and its sequence of application [2–7]. More specifically, which should be utilized first: PP or angioembolization? Those who support the application of PP before angioembolization reason that PP controls the primary sources of bleeding, venous and fractured cancellous bones, that time to PP is faster than time to angioembolization, associated injuries can be simultaneously treated, and that PP first allows for determination of the source of bleeding and thereby the need for angioembolization [2, 8–10]. Whereas those in favor of angioembolization first contend that PP may only be necessary at trauma centers with slower intervention preparation times for interventional radiology (IR), PP is more invasive, PP requires a second invasive operation to remove the packs, and PP increases the risk for infection [2].

Time to angioembolization may be longer than time to PP due to the time it takes for IR to prepare for angioembolization for a hemodynamically unstable pelvic fracture. The American College of Surgeons (ACS) guidelines for Level I trauma centers state that the operating room must be promptly available for emergency operations on musculoskeletal injuries, including all the necessary resources, and additionally recommend a designated orthopaedic fracture room [11], whereas the ACS does not require the IR team to be prepared for such patients [11]. Theoretically, a patient with a hemodynamically unstable pelvic fracture could be admitted to the operating room without delay for immediate operation but could potentially have to wait for IR to prepare the room for intervention. Similarly, orthopaedic surgeons and interventional radiologists are not required to work on-site but must be available in within 30 minutes (min.) per the ACS guidelines for Level I trauma centers [11]. We previously reported that a majority (71%) of Level I trauma centers surveyed indicated an arrival time of 21–30 min when interventional radiologists worked off-site [12]. Additionally, 54% of Level I trauma centers surveyed had 24-h/day on-site interventional radiologist coverage, with the remaining sites having on-call coverage [12]. Minimizing the time between arrival and definitive bleeding control has shown to improve outcomes and is therefore recommended by the World Society of Emergency Surgeons [3, 9, 13]. To our knowledge, there have been no studies that compared the availability of orthopaedic surgeons to the availability of interventional radiologists to treat patients with hemodynamically unstable pelvic fractures; this data could contribute to the development of the optimal guideline on the use PP or angioembolization first, as a shorter time to hemorrhage control can improve outcomes.

Although the orthopaedic trauma department at Level I trauma centers must be overseen by an individual who has completed a fellowship in orthopaedic traumatology, there are no staffing requirements from the ACS on the number of orthopaedic surgeons trained in pelvic fracture management [11]. Previous studies have found that the addition of orthopaedic surgeons trained to manage pelvic fractures was related to both the proportion of patients undergoing definitive operative repair and a decrease in mortality rates for patients with pelvic fractures [14, 15]. Because of the lower mortality rates among hospitals with an orthopaedic surgeon trained to manage pelvic fractures, it is important to determine if there is a relationship between the priority to use PP or angioembolization first and the number of fellowship-trained traumatologists specializing in orthopaedics with or without *training to manage pelvic fractures.*

The study hypotheses were that (1) the orthopaedic surgeons and interventional radiologists would have significant differences in coverage at Level I trauma centers, and (2) the priority to use PP or angioembolization first would be dependent on: the number of fellowship-trained traumatologists specializing in orthopaedics with or without *training to manage pelvic fractures*, the time for orthopaedic surgeons or interventional radiologists to arrive when working off-site, and the time for orthopaedic surgeons to respond to a consultation call.

## Methods and materials

The Western Institutional Review Board approved this anonymous cross-sectional survey. Trauma medical directors at all 158 United States ACS-verified Level I trauma centers were invited to participate, to view the invitation list, visit http://bit.ly/TraumaCenterInvites. Six email invitations, containing the approved partial waiver of consent requiring no signature, were distributed through SurveyMonkey Inc. (San Mateo, CA; www.surveymonkey.com) from March 2018 to June 2018. Before the fifth and sixth survey invitations were sent, trauma medical directors were called to verify receival of the survey invitation. There was no compensation provided for participation, which was voluntary. “Participants” are trauma medical directors or an assigned colleague who completed the survey.

Co-authors drafted the 46-question survey, and two trauma medical directors piloted the survey. Questions pertaining to this paper are available at http://bit.ly/OrthopedicSurveyQuestions. Questions were asked about the availability orthopaedic surgeons and interventional radiologists, the number and availability of fellowship-trained traumatologists specializing in orthopaedics with or without *training to manage pelvic fractures*, and the treatment sequence for hemodynamically unstable pelvic fractures. A feature of SurveyMonkey called “skip logic” was used to skip irrelevant questions based on previous closed-ended responses. Participants could also skip any question for any reason; therefore, there are missing responses for various individual questions. The study aims were to (1) compare the coverage of orthopaedic surgeons and interventional radiologists at Level I trauma centers, and (2) determine if the number of fellowship-trained traumatologists specializing in orthopaedics with or without *training to manage pelvic fractures*, the time for orthopaedic surgeons or interventional radiologists to arrive when working off-site, and the time for orthopaedic surgeons to respond to a consultation call affected the priority to use PP or angioembolization first for hemodynamically unstable pelvic fractures.

SurveyMonkey’s skip logic skipped two questions on (1) time to arrive when working off-site and (2) the number of hours per day with on-call coverage for participants who indicated they had 24-h/day coverage. Participants with 24-h/day coverage were designated as having 0 h/day with on-call coverage and 0 min to arrive when working off-site. Fellowship-trained traumatologists specializing in orthopaedics (without training to manage pelvic fractures) will be referred to as orthopaedic trauma surgeons. Fellowship-trained traumatologists specializing in orthopaedics *trained to manage pelvic fractures* will be referred to as orthopaedic trauma surgeons *trained to manage pelvic fractures*. The median number of orthopaedic trauma surgeons and the mean number of orthopaedic trauma surgeons *trained to manage pelvic fractures* were used to categorize participating Level I trauma centers into two groups: those with above the median or mean and those with equal to or below the median or mean. Questions on orthopaedic surgeon’s coverage, time to arrive, and time to respond to a consultation were regarding all orthopaedic surgeons regardless of training status. Analyses on if PP or angioembolization was utilized first included responses only from participants who utilized both angioembolization and PP.

SAS 9.4 (Cary, NC) software was used for statistical analyses. Descriptive data were expressed as counts (percentages), mean (standard deviation), or median (interquartile range). Fisher’s exact test, chi-squared, and Kruskal-Wallis test were used, with an alpha of 0.05.

## Results

Of the 158 trauma medical directors invited to participate, 25% (40) responded to the survey; of those 90% (36/40) completed the survey and 10% (4/40) partially completed the survey. All survey responses were included. The survey took a median (IQR) of 11 min (8–21) for those that completed the entire survey. The most common response for the U.S. Census Bureau region was the South (40% [16/40]) [16]. A majority of participants (90% [36/40]) had more than 1501 trauma patients in 2017 and 58% (23/40) had been an ACS-verified Level I trauma center for over 10 years. More details on the characteristics of the participating Level I trauma centers have been reported [17].

Table 1 displays questions and responses pertaining to the orthopaedic surgeons and their availability. Most participating Level I trauma centers had orthopaedic surgeons on-site 24-h/day, and all orthopaedic surgeons arrived within 30 min of patient arrival when working off-site. Most participants reported a response time of 0 to 10 min after receiving a consultation call for a hemodynamically unstable patient with a pelvic fracture for orthopaedic surgeons. The participating trauma centers had an average (SD) of 3 (2) orthopaedic surgeons *trained to manage pelvic fractures* and a median (IQR) of 0 (0–0) orthopaedic trauma surgeons (without training to manage pelvic fractures). A majority (71% [25/35]) of participating Level I trauma centers had orthopaedic trauma surgeons *trained to manage pelvic fractures* available 7 days/week. Of those who did not have coverage 7 days/week, only one participant (10% [1/10]) responded that the orthopaedic trauma surgeons *trained to manage pelvic fractures* were not available within 24 h of patient arrival.

**Table 1:** Survey questions and responses

	% (*n*)	*n*
Does the orthopaedic department have 24-h/day in-hospital coverage?		
Yes	86% (31)	36
No	14% (5)	
How many hours per day is there only an on-call orthopaedic surgeon available?^a^		
8 h	40% (2)	5
10 h	20% (1)	
24 h	40% (2)	
Approximately how long does it take for an orthopaedic surgeon to arrive when working off-site?		
0–10 min	0	5
11–20 min	20% (1)	
21–30 min	80% (4)	
≥ 31 min	0	
How many orthopaedic trauma surgeons *trained to manage pelvic fractures* are employed at your hospital?		
0	3% (1)	35
1	17% (6)	
2	11% (4)	
3	26% (9)	
4	20% (7)	
5	14% (5)	
6	6% (2)	
7	0	
8	3% (1)	
9	0	
10 or more	0	
Mean (SD)	3 (2)	
How many orthopaedic trauma surgeons are employed at your hospital? (without training to manage pelvic fractures)		
0	83% (29)	35
1	11% (4)	
2	3% (1)	
3	0	
4	0	
5	0	
6	3% (1)	
7	0	
8	0	
9	0	
10 or more	0	
Median (IQR)	0 (0, 0)	
How many days per week are there available fellowship-trained traumatologists specializing in orthopaedics and trained to manage pelvic fractures?^b^		
5 days	23% (8)	35
6 days	6% (2)	
7 days	71% (25)	
Are the fellowship-trained traumatologists specializing in orthopaedics and trained to manage pelvic fractures available within 24 h of the patient’s arrival?		
Yes	90% (9)	10
No	10% (1)	
Approximately how long does it take for the orthopaedic department to respond to a consultation call for a hemodynamically unstable patient with a pelvic fracture?^c^		
0–10 min	44% (16)	36
11–20 min	22% (8)	
21–30 min	33% (12)	
≥ 31 min	0	
Who was faster to arrive?^c^ % (*n*)		
Interventional radiologists	6% (2)	36
Orthopaedic surgeons	39% (14)	
Arrival times reported are equal	56% (20)	

^a^Participants could select any number of hours from 0 to 24

^b^Participants could select any number of days from 0 to 7

^c^Percentages may total more or less than 100% due to rounding

The orthopaedic surgeons were on-site 24-h/day more often than interventional radiologists, 86% versus 54%, respectively, *p* = 0.003 (Table 2). At Level I trauma centers without 24-h/day coverage, interventional radiologists took longer to arrive than the orthopaedic surgeons; no participants reported an arrival time greater than 30 min for orthopaedic surgeons, whereas 11% (4/35) of participants reported that the interventional radiologists took equal to or greater than 31 min to arrive, *p* = 0.006. The odds of having on-site coverage 24-h/day were 5.3 (1.7, 16.6) times higher for orthopaedic surgeons than for interventional radiologists, *p* = 0.004. The odds were 1.4 (0.9, 2.0) times higher that the participants reported a higher number of hours per day with on-call coverage for the interventional radiologists, when compared to on-call coverage for the orthopaedic surgeons; however, this was not significant, *p* = 0.12.

**Table 2:** Orthopaedic surgeon and interventional radiologist coverage at US level 1 trauma centers

	Orthopaedic surgeons	Interventional radiologists	*p*
24-h/day on-site coverage % (*n*)			
Yes	86% (31)	54% (20)	0.003
No	14% (5)	46% (17)	
OR (95% CI)	Ref.	5.3 (1.7, 16.6)	0.004
Time to arrive % (*n*)			
0 min^a^	86% (31)	54% (20)	0.006
0–10 min	0	0	
11–20 min	3% (1)	3% (1)	
21–30 min	11% (4)	32% (12)	
≥ 31 min	0	11% (4)	
On-call coverage per day in hours			
Median (IQR)	0 (0–0)	0 (0–12)	0.005
OR (95% CI)	Ref.	1.4 (0.9, 2.0)	0.12
Who was faster to arrive and treatment prioritization			
Angioembolization first	63% (5)	100% (2)	> 0.99
Pelvic packing first	37% (3)	0% (0)	

*P p* value, *IQR* interquartile range, *OR* odds ratio, *CI* confidence interval

^a^Participants who had on-site 24-h/day coverage

A majority (56% [20/36]) of the participants reported the same arrival time for interventional radiologists and orthopaedic surgeons (Table 1). There were more participants (39% [14/36]) who reported that the orthopaedic surgeons arrived faster than those who reported that the interventional radiologists were faster to arrive. Only two participants (6% [2/36]) reported that the interventional radiologists arrived faster than the orthopaedic surgeons. The prioritization of angioembolization or PP first was not significantly associated with the faster arrival of orthopaedic surgeons or interventional radiologists (Table 2). Both of the Level I trauma centers having faster arrival time for interventional radiologists (100% [2/2]) utilized angioembolization first, whereas 37% (3/8) of the Level I trauma centers that had a faster arrival time for orthopaedic surgeons used PP first.

The proportion of participating Level I trauma centers that utilized angioembolization before PP has been reported, a majority (63% [17/27]) of participants reported that angioembolization was prioritized before application of PP, and 37% (10/27) applied PP before angioembolization [17]. The prioritization of PP before angioembolization was examined according to (1) orthopaedic surgeon’s arrival time, (2) interventional radiologist’s arrival time, (3) orthopaedic surgeon’s time to respond to a consultation call, and (4) interventional radiologist’s time to prepare for intervention (Table 3). Our data shows that none of the timing metrics for interventional radiologists or orthopaedic surgeons were significantly associated with the proclivity to utilize angioembolization or PP first.

**Table 3:** Angioembolization or pelvic packing first given physician availability

	Pelvic packing first	Angioembolization first	*p*
Orthopaedic surgeon’s time to arrive % (*n*)			
0 min^a^	41% (9)	59% (13)	> 0.99
0–10 min	0	0	
11–20 min	0	100% (1)	
21–30 min	25% (1)	75% (3)	
≥ 31 min	0	0	
Intervention radiologist’s time to arrive % (*n*)			
0 min^a^	38% (6)	63% (10)	0.54
0–10 min	0	0	
11–20 min	100% (1)	0	
21–30 min	25% (2)	75% (6)	
≥ 31 min	50% (1)	50% (1)	
Orthopaedic surgeon’s time to respond to consultation % (*n*)			
0–10 min	50% (6)	50% (6)	0.60
11–20 min	29% (2)	71% (5)	
21–30 min	25% (2)	75% (6)	
Intervention radiologists time to prepare for intervention % (*n*)			
0–30 min	36% (5)	64% (9)	> 0.99
31–60 min	33% (3)	67% (6)	
61–120 min	50% (2)	12% (2)	
Number of orthopaedic trauma surgeons % (*n*)			
Above median (> 0)	0% (0)	100% (6)	0.06
Equal to median (= 0)	48% (10)	52% (11)	
Number of orthopaedic trauma surgeons *trained to manage pelvic fractures* % (*n*)			
Above average (> 3)	62% (8)	38% (5)	0.02
Equal to or below average (≤ 3)	14% (2)	86% (12)	

*Ref* reference, *OR* odds ratio, *CI* confidence interval, *p p* value

^a^Participants who had on-site 24-h/day coverage

All Level I trauma centers with above the median number (> 0) of orthopaedic trauma surgeons prioritized angioembolization before PP, whereas 48% of Level I trauma centers with equal to the median number (= 0) of orthopaedic trauma surgeons prioritized PP before angioembolization, but this was not significant (Table 3). However, having above the average number orthopaedic trauma surgeons *trained to manage pelvic fractures* did significantly affect the prioritization of PP before angioembolization. A majority [62% (8)] of Level I trauma centers with above the average number of orthopaedic trauma surgeons *trained to manage pelvic fractures* (> 3) utilized PP first, whereas a majority [86% (12)] of Level I trauma centers with equal to or below the average number (≤ 3) of orthopaedic trauma surgeons *trained to manage pelvic fractures* utilized angioembolization first, *p* = 0.02.

## Discussion

Previous studies have found that time to angioembolization is longer than time to PP, which may be in part due to the findings from this study on the availability of the orthopaedic surgeons compared to the interventional radiologists [8, 10, 18, 19]. Orthopaedic surgeons were on-site 24-h/day more often than interventional radiologists. At Level I trauma centers without 24-h/day coverage, orthopaedic surgeons had less hours per day with on-call coverage only. Survey participants also reported faster arrival times for orthopaedic surgeons when compared to interventional radiologists. Furthermore, the interventional radiologists must prepare for intervention for a hemodynamically unstable patient with a pelvic fracture. We previously reported that 46% of participating Level I trauma centers indicated intervention preparation times between 31 and 120 min [12], whereas the ACS guidelines require Level I trauma centers to have an operating rooms promptly available to allow for emergency operations on musculoskeletal injuries, such as pelvic fractures [11]. Therefore, not only are orthopaedic surgeons available on-site more often and faster to arrive, but the operating rooms are also prepared to manage a hemodynamically unstable patient with a pelvic fracture, whereas interventional radiologists must prepare for angioembolization. Given that mortality rates are lower when time from arrival to hemorrhage control is reduced, it may be advantageous for PP to be utilized before angioembolization; however, outcome data is needed to confirm this [3, 9, 13].

Unexpectedly, this study found no association between the time to arrive or time to respond to a consultation call for a hemodynamically unstable pelvic fracture for orthopaedic surgeons, the time to arrive or time to prepare for intervention for interventional radiologists, and the prioritization of PP before angioembolization. This is similar to previous findings that the time to arrive and time to prepare for intervention for the interventional radiologists did not affect the priority treatment sequence for angioembolization for hemodynamically unstable pelvic fractures [12]. This further suggests that the priority treatment sequence for hemodynamically unstable pelvic fractures is not dependent on the availability of neither interventional radiologists nor orthopaedic surgeons.

However, this study did find an association between the number of orthopaedic trauma surgeons *trained to manage pelvic fractures* and the prioritization of PP application before the use of angioembolization. Perkins et al. found that having two orthopaedic surgeons dedicated to pelvic trauma resulted in an increase in the proportion of patients undergoing definitive operative repair of unstable pelvic trauma, *p* = 0.004, with a significant decrease in mortality rates after appointment of the dedicated pelvic orthopaedic surgeons, *p* = 0.025 [14]. Another study by Biffl et al. observed mortality rates before and after the modification of a multidisciplinary clinical pathway for pelvic fractures with the addition of two orthopaedic pelvic trauma specialists found the post-group had a 16% lower mortality rate, *p* < 0.05 [15]. The results of this survey also show differences in treatment based on the number of orthopaedic trauma surgeons *trained to manage pelvic fractures*. Level I trauma centers that had above the average number of orthopaedic trauma surgeons *trained to manage pelvic fracture* prioritized the application of PP before the use of angioembolization more often than those with equal to or below average. Although data on the number of interventional radiologists staffed at Level I trauma centers was not collected, it is possible that there are more interventional radiologists than orthopaedic surgeons staffed at Level I trauma centers. Glassdoor reports that the average pay for an interventional radiologist is roughly $90,000 less expensive than the average pay for orthopaedic surgeons [20, 21]. This could be contributing to the overall tendency towards utilizing angioembolization first; however, more data is needed to confirm this.

### Limitations

This study surveyed trauma medical directors at ACS-verified Level I trauma centers in the United States, with a response rate of 25%. Given the small number of US Level I trauma centers, a response rate of 25% only included 40 Level I trauma centers, and not all participants completed the survey. Furthermore, mortality data at individual Level I trauma centers was not collected, so conclusions could not made if any treatment sequences were superior in terms of mortality rates. The availability of trauma surgeons and general surgeons also was not collected. It is possible that trauma or general surgeons would pack the pelvis rather than waiting for an orthopaedic surgeon to arrive. Therefore, there are more departments available that can apply PP than there are that can conduct angioembolization; had data also been collected on the availability of trauma surgeons and general surgeons, there may have been an association between the availability of surgeons who can apply PP and the priority treatment sequence. Additionally, the number of interventional radiologists hired was not collected and may play a role in the decision to use angioembolization first. Self-report and recall bias could have impacted responses; survey anonymity and instructions to have pelvic fracture management protocols on-hand were precautions to reduce these biases. The participant responses to this survey also may not be depictive of all Level I trauma centers.

## Conclusions

Our data provides evidence that the orthopaedic surgeons were more likely to be on-site 24-h/day than the interventional radiologists at Level I trauma centers. Additionally, the orthopaedic surgeons were often faster to arrive than the interventional radiologists. Despite this and contrary to anecdotal evidence that PP would be utilized before angioembolization due to the availability of orthopaedic surgeons, there was a lack of association between the prioritization of PP over angioembolization and who was faster to arrive: interventional radiologists or orthopaedic surgeons. Level I trauma centers should review the availability of both interventional radiologists and orthopaedic surgeons, published treatment guidelines, and current time to hemorrhage control for PP and angioembolization, to determine if they are utilizing their resources in an optimal treatment sequence to prevent mortality. Additionally, the number of orthopaedic trauma surgeons *trained to manage pelvic fractures* was associated with the utilization of PP or angioembolization first. There was a significantly higher proportion of Level I trauma centers with more than the average number (> 3) of orthopaedic trauma surgeons *trained to manage pelvic fractures* who utilized PP before angioembolization than Level I trauma centers with equal to or below the average number of orthopaedic trauma surgeons *trained to manage pelvic fractures*. However, more data is needed to determine how the addition of orthopaedic trauma surgeons *trained to manage pelvic fractures* affects patient outcomes. As pelvic fracture management is a multidisciplinary approach often dependent on the presenting patient’s injuries, we suggest adequate 24-h staffing of both orthopaedic trauma surgeons and interventional radiologists to treat patients on a case-by-case basis with any treatment type deemed appropriate.

## Data Availability

Data for this study is stored on Sharefile, an electronic HIPAA and HITECH-compliant platform that ensures all transmissions are fully encrypted, end-to-end. The datasets used for analysis for the current study are available from the corresponding author on reasonable request.

## Abbreviations

ACS: American College of Surgeons
IR: Interventional radiology
PP: Pelvic packing
US: United States
